# Supplementary material for: Multimodal single cell sequencing implicates chromatin accessibility and genetic background in diabetic kidney disease progression
Source: Nat Commun. 2022 Sep 6;13:5253. doi: 10.1038/s41467-022-32972-z (PMC9448792; doi:10.1038/s41467-022-32972-z)
Supplement: Supplementary file 1 — Supplementary Information [file 41467_2022_32972_MOESM1_ESM.pdf]

Supplementary Information

Multimodal single cell sequencing implicates chromatin accessibility and genetic background in diabetic kidney disease progression

Parker C. Wilson, Yoshiharu Muto, Haojia Wu, Anil Karihaloo, Sushrut S. Waikar, and Benjamin D. Humphreys

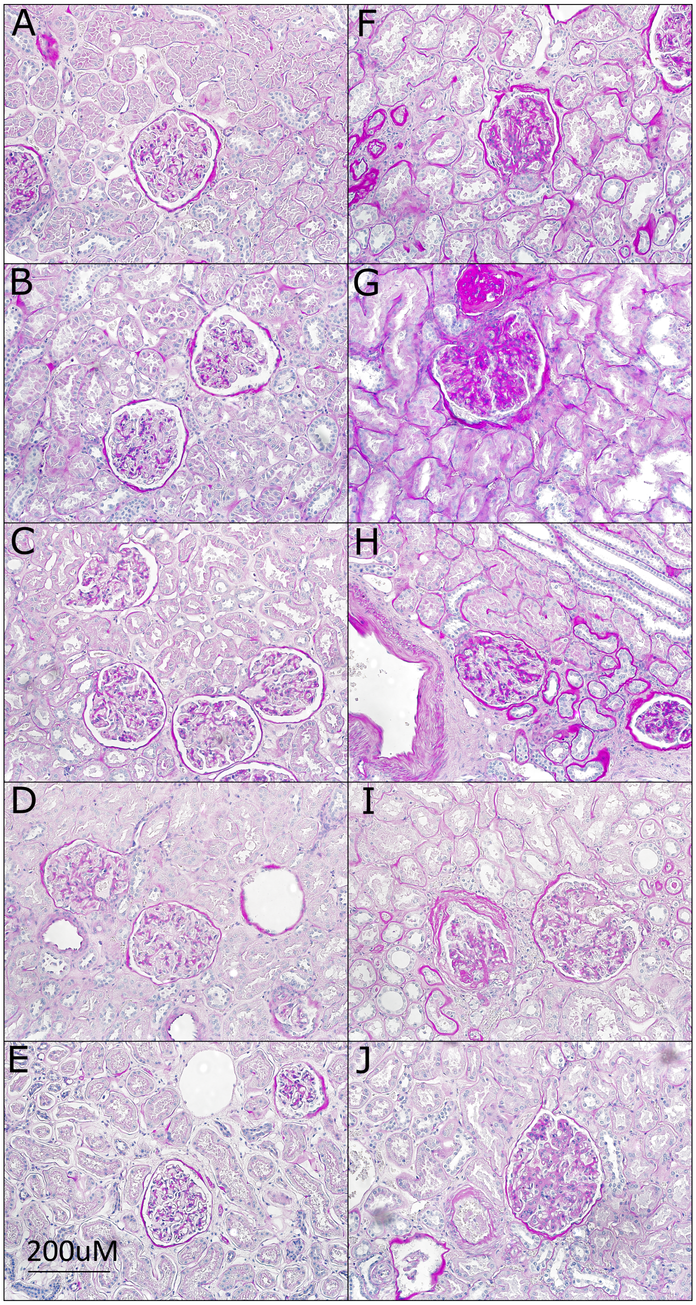

Supplemental Figure 1: Representative Histology of Control and DKD Samples (PAS 200X). A-E) Control samples 1-5 showing normal glomerular and tubulointerstitial histology without evidence of glomerular basement membrane thickening, mesangial matrix expansion, Bowman's capsule fibrosis, interstitial fibrosis or tubular atrophy **F-J)** DKD samples 1-5 showing glomerular basement membrane thickening with mesangial matrix expansion in association with tubular atrophy and vascular disease. A total of 10 images were taken for each donor sample with similar results.

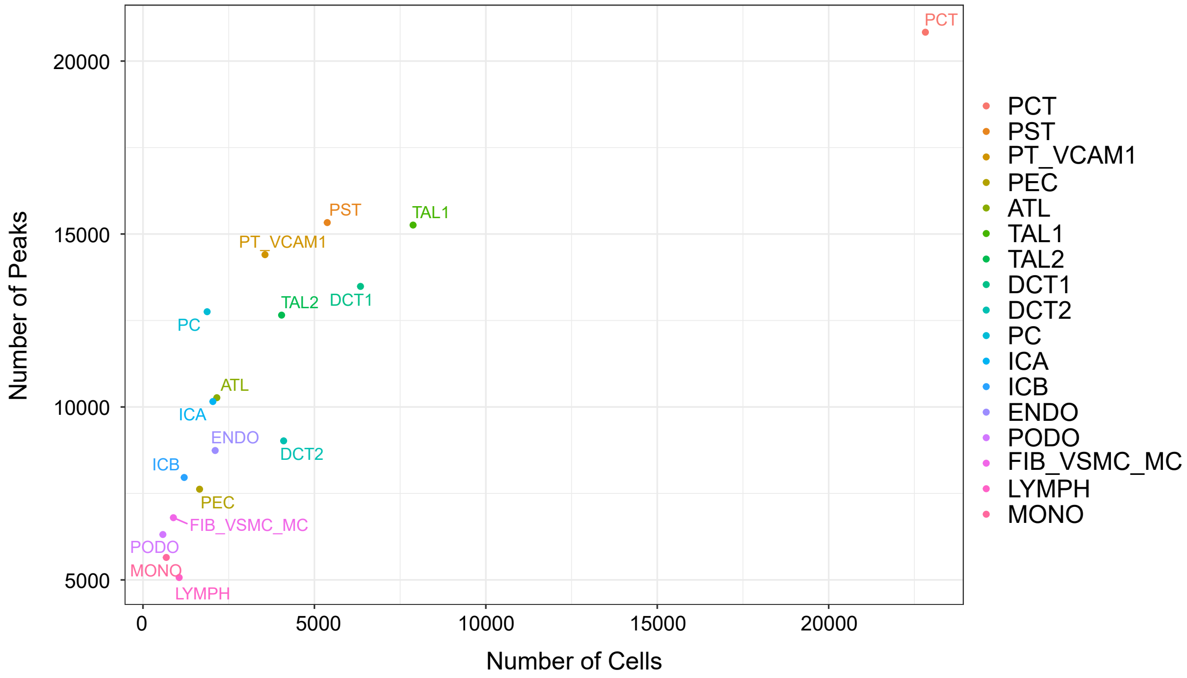

Supplemental Figure 2. Number of MACS2 ATAC peaks called for each cell type. Individual snATAC-seq cellranger-atac counts were aggregated for all thirteen libraries, preprocessed, and filtered with Signac. Cell-specific ATAC peaks were called separately for each cell type using MACS2 and the Signac wrapper function. N=13 biologically independent samples containing 68,458 cells were evaluated in a joint analysis. Significance was determined using a Benjamini-Hochberg adjusted Poisson-distribution p-value based on lambda. The number of cell-specific ATAC peaks meeting the adjusted p-value threshold (padj < 0.05) and the total number of cells passing quality control filters are visualized in a scatter plot.

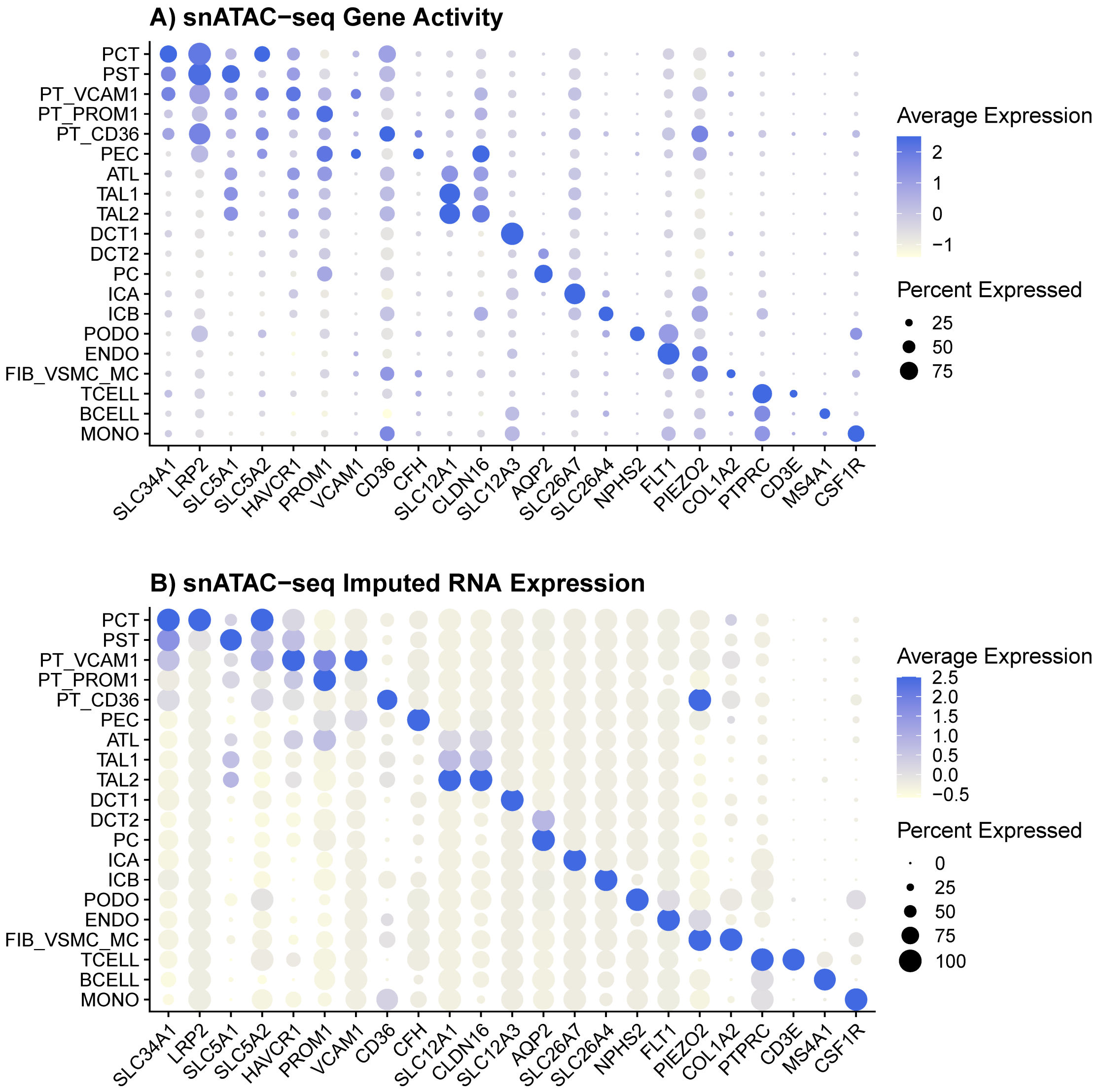

Supplemental Figure 3. Lineage-specific markers in snATAC-seq. A) Gene activity was computed for the aggregated and preprocessed snATAC-seq object for thirteen libraries using the gene body and promoter region of protein-coding genes and visualized with the Seurat DotPlot function. **B)** The aggregated snRNA-seq object was coembedded with the snATAC-seq object and estimated RNA expression values for the snATAC-seq object were computed using label transfer. Imputed RNA expression values are visualized with the Seurat DotPlot function.

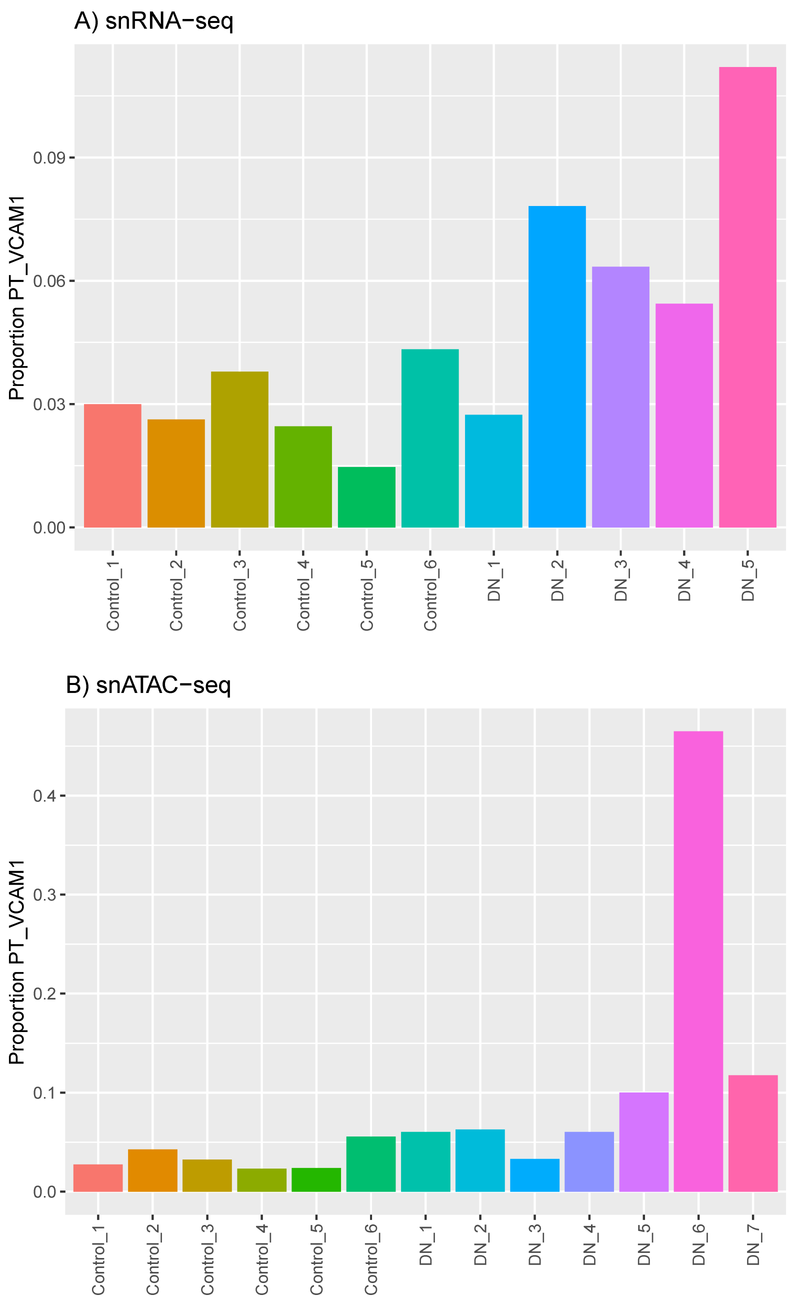

Supplemental Figure 4: Proportion of PT_VCAM1 across donors by snRNA-seq and snATAC-seq. A) For the aggregated snRNA-seq object, the proportion of cells annotated as PT_VCAM1 was divided by the total number of cells per donor. **B)** For the aggregated snATAC-seq object, the proportion of cells annotated as PT_VCAM1 was divided by the total number of cells per donor

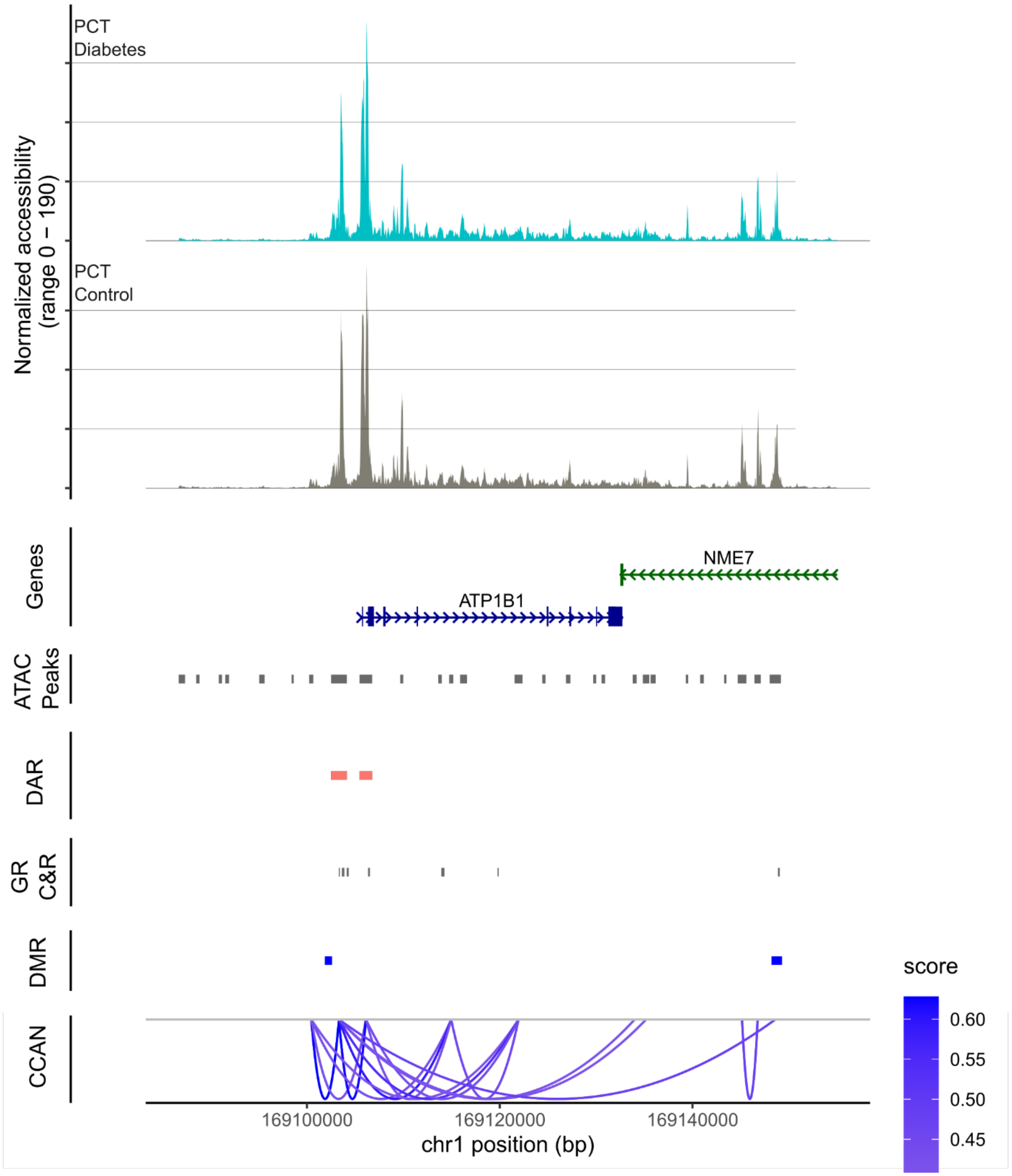

Supplemental Figure 5: Proximal tubule-specific DAR and ATAC peaks in *ATP1B1*. snATAC-seq coverage plots for DKD and control PCT are displayed in relation to the gene body. (ATAC Peaks) – accessible ATAC peaks in PCT. (DAR) – cell-specific DAR in diabetic PCT (Supplemental Data 3). (GR C&R) – bulk kidney GR CUT&RUN binding sites. (DMR) – differentially methylated regions (Supplemental Data 19). (CCAN) - Blue arcs depict the nodes of a cis-coaccessibility network (CCAN) surrounding the gene body.

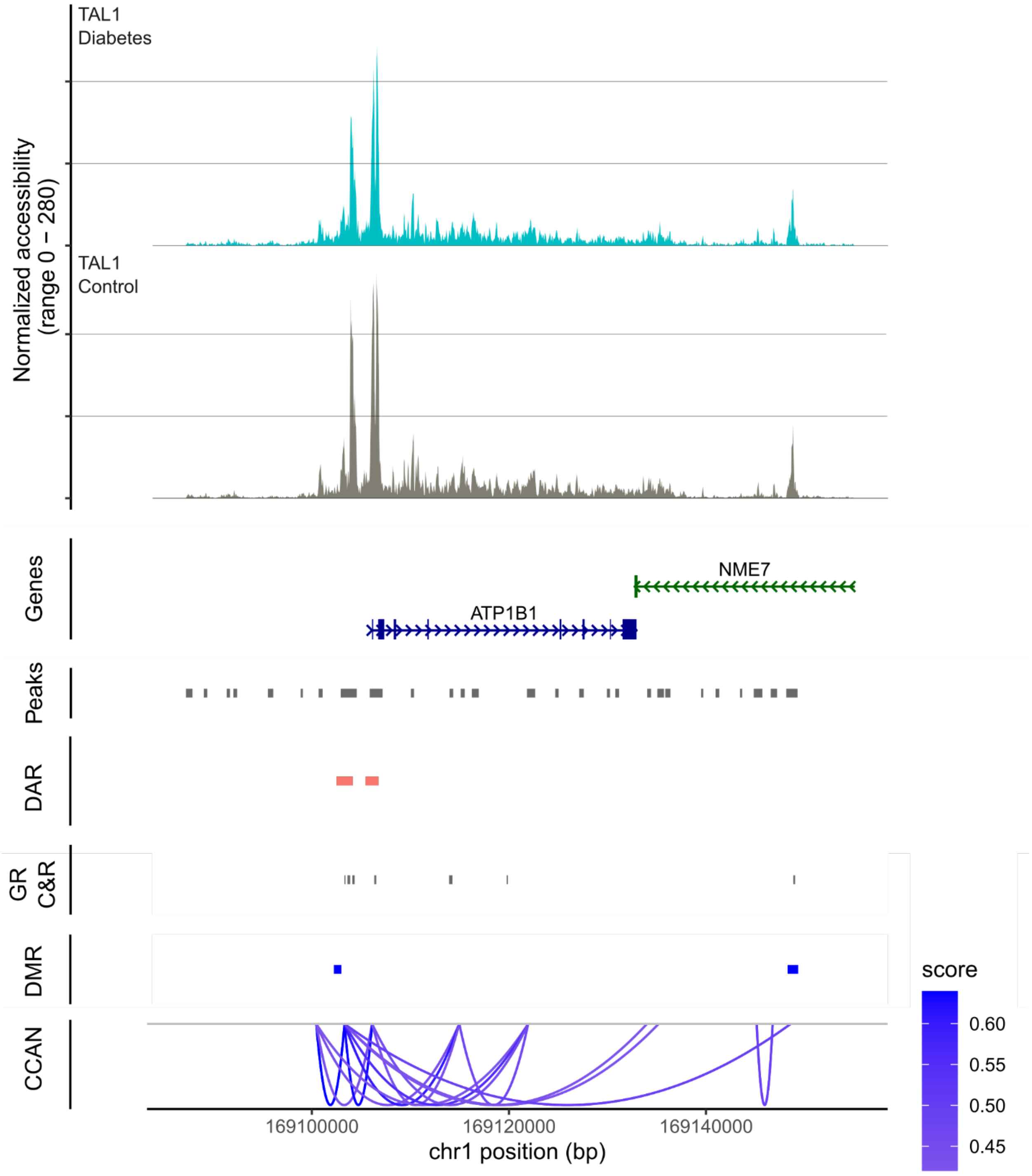

Supplemental Figure 6: Thick Ascending Limb DAR and ATAC peaks in *ATP1B1*. snATAC-seq coverage plots for DKD and control TAL1 are displayed in relation to the *ATP1B1* gene body. (ATAC Peaks) – accessible ATAC peaks in TAL1. (DAR) – cell-specific DAR in diabetic TAL1 (Supplemental Data 3). (GR C&R) – bulk kidney GR CUT&RUN binding sites. (DMR) – differentially methylated regions (Supplemental Data 19). (CCAN) - Blue arcs depict the nodes of a cis-coaccessibility network (CCAN).

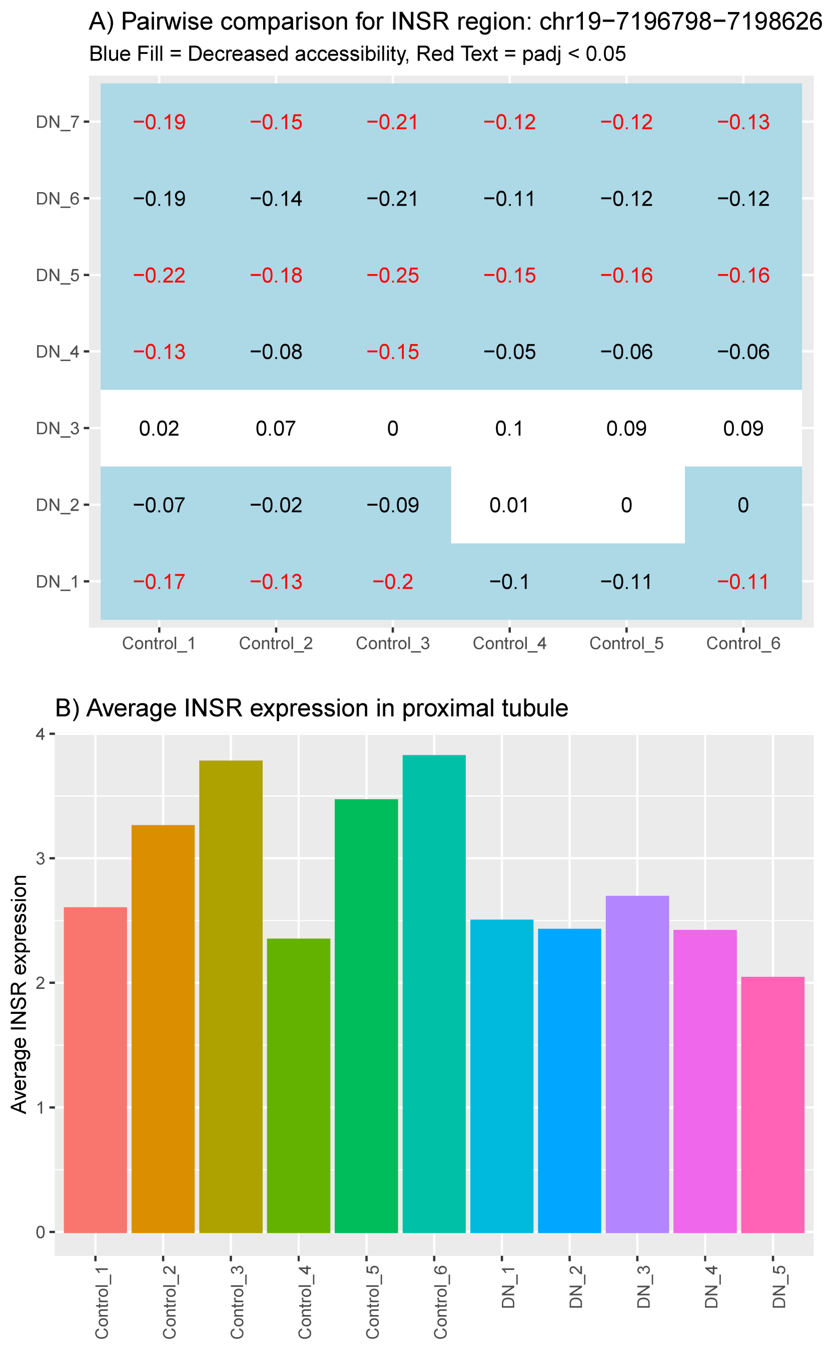

Supplemental Figure 7: Differential accessibility and expression of *INSR* in proximal tubule. A) Pairwise comparisons between each control and DKD donor for the proximal convoluted tubule (PCT) were made for the *INSR* DAR chr19:7196798-7198626 using the aggregated snATAC-seq dataset. Comparisons that show decreased accessibility in DKD relative to control (avg_log2FC < 0) are filled blue and comparisons that have an adjusted pval < 0.05 (Bonferroni-adjusted Wilcoxon Rank Sum) have red text. **B)** Average *INSR* expression in the proximal tubule (PT, PTVCAM1) in the aggregated snRNA-seq dataset was calculated using the Seurat AverageExpression function and the RNA assay.

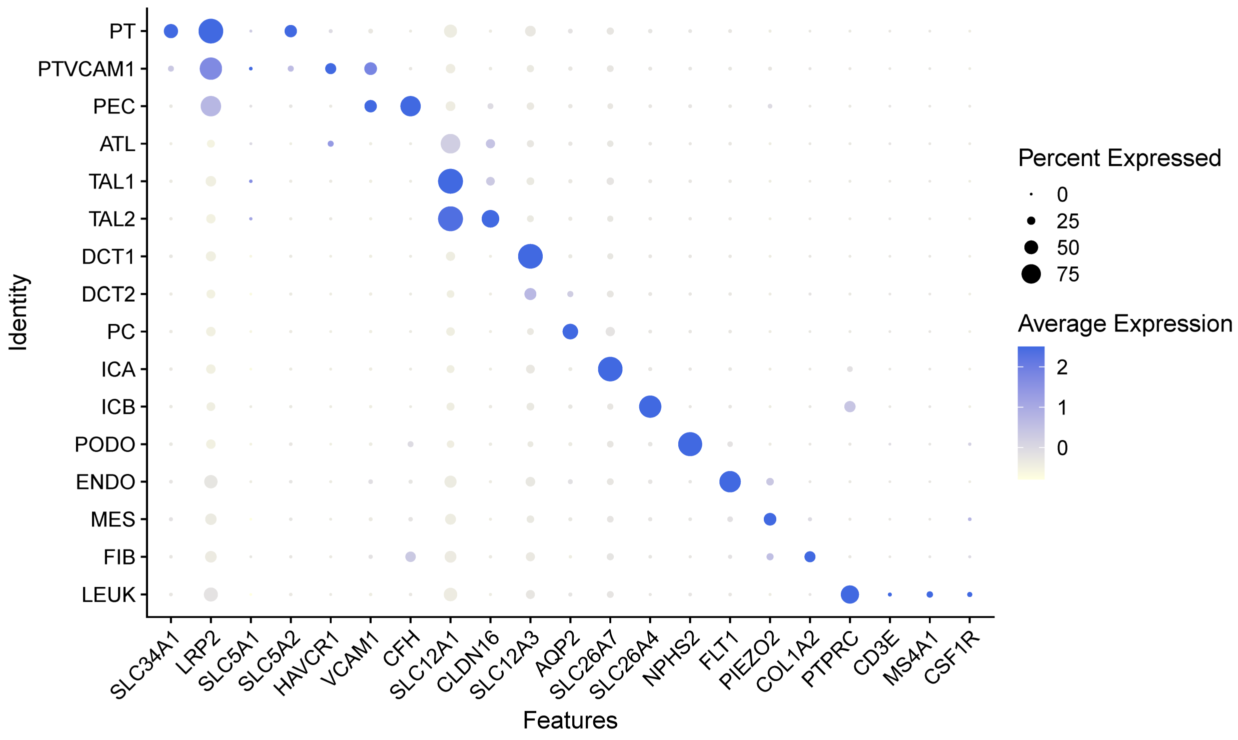

Supplemental Figure 8. Lineage-specific markers by snRNA-seq. Individual snRNA-seq libraries were aggregated, preprocessed, and filtered with Seurat. Lineage-specific markers for normalized RNA expression of individual cell types are visualized using the Seurat DotPlot function.

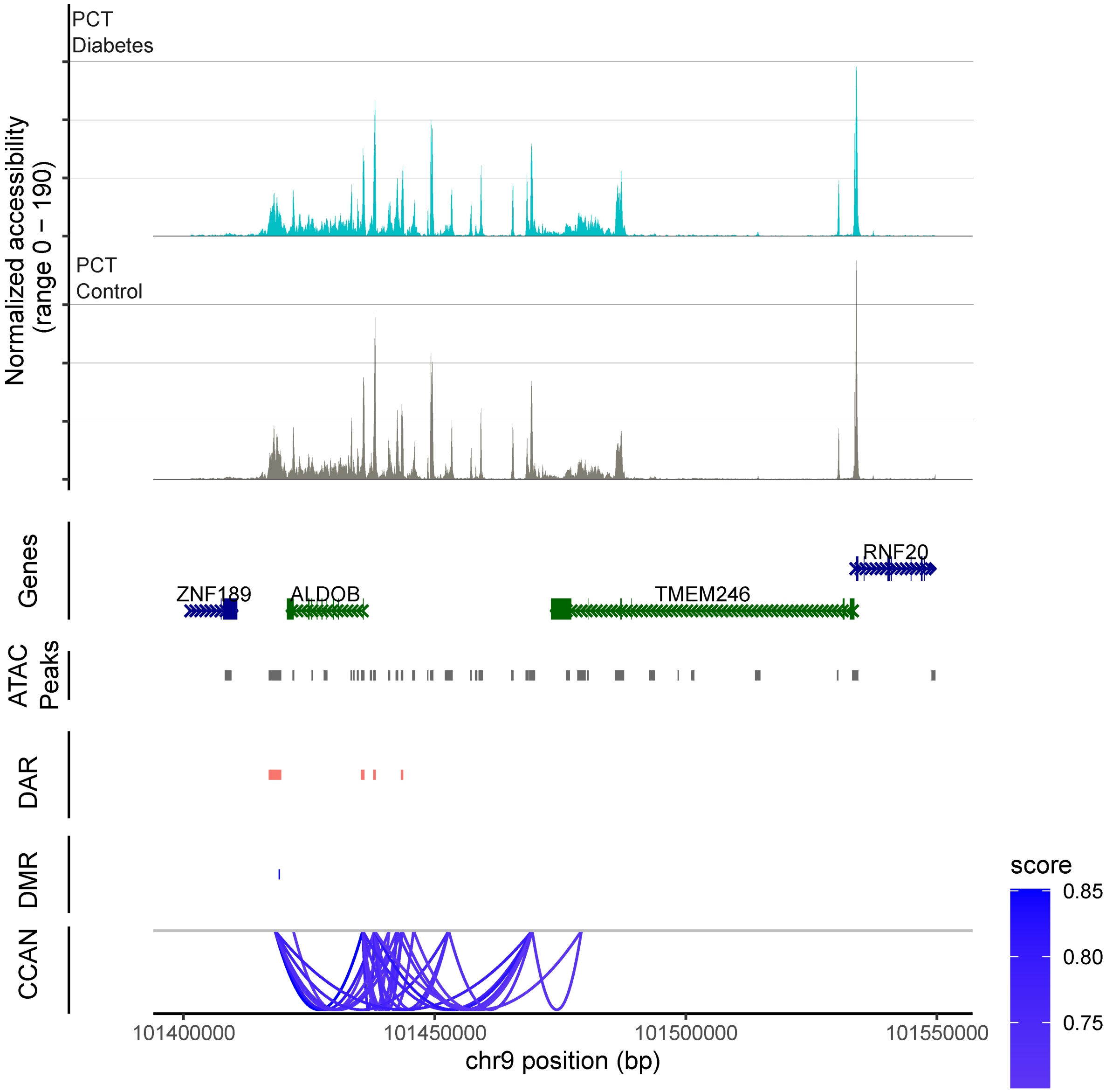

Supplemental Figure 9: Proximal tubule-specific DAR and ATAC peaks in *ALDOB*. snATAC-seq coverage plots for DKD and control PCT are displayed in relation to the *ALDOB* gene body. (ATAC Peaks) – accessible ATAC peaks in PCT. (DAR) – cell-specific DAR in diabetic PCT (Supplemental Data 3). (DMR) – differentially methylated regions (Supplemental Data 19). (CCAN) - Blue arcs depict the nodes of a cis-coaccessibility network (CCAN).

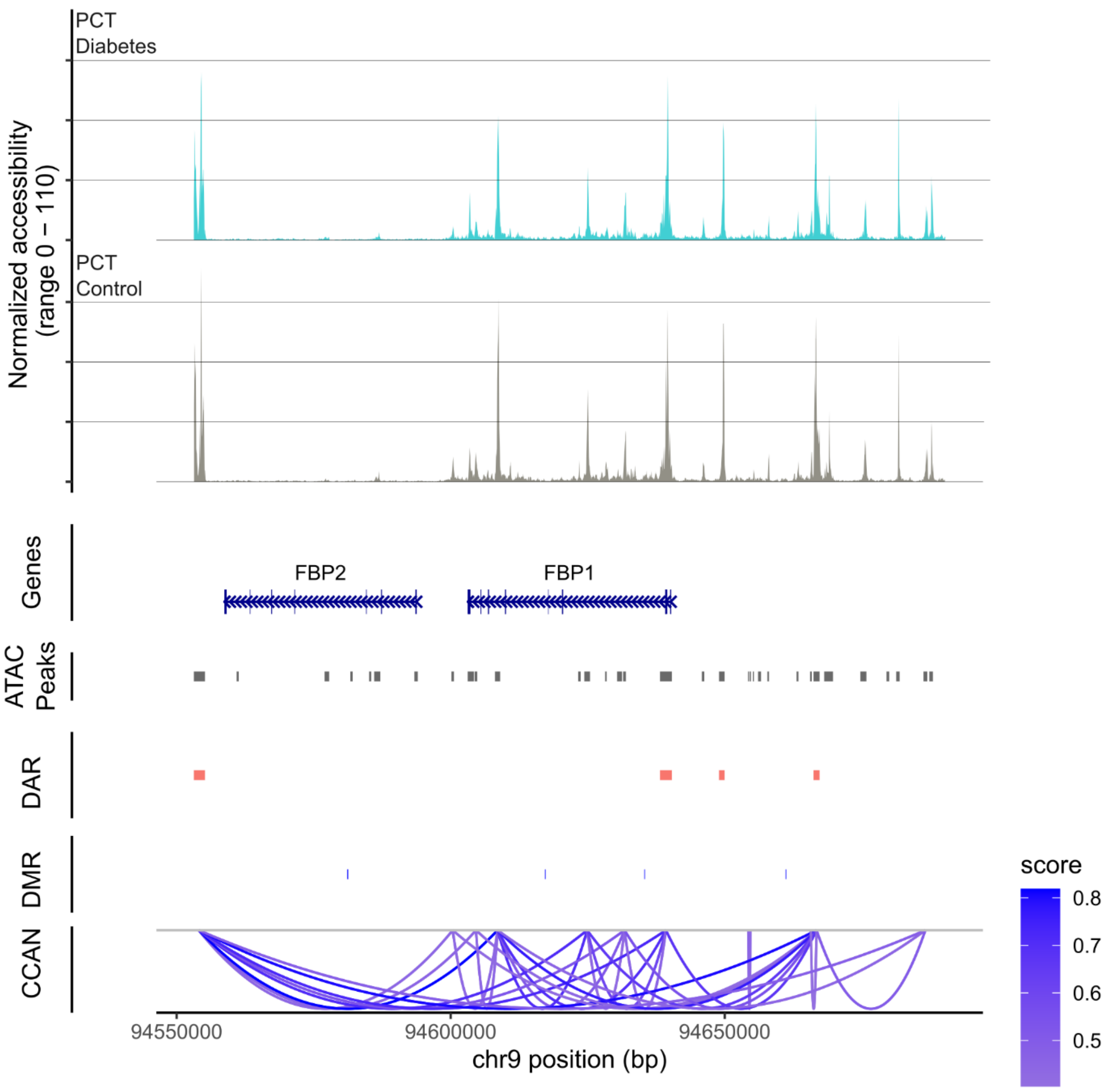

Supplemental Figure 10: Proximal tubule-specific DAR and ATAC peaks in *FBP1*. snATAC-seq coverage plots for DKD and control PCT are displayed in relation to the *FBP1* gene body. (ATAC Peaks) – accessible ATAC peaks in PCT. (DAR) – cell-specific DAR in diabetic PCT (Supplemental Data 3). (DMR) – differentially methylated regions (Supplemental Data 19) (CCAN) - Blue arcs depict the nodes of a cis-coaccessibility network (CCAN).

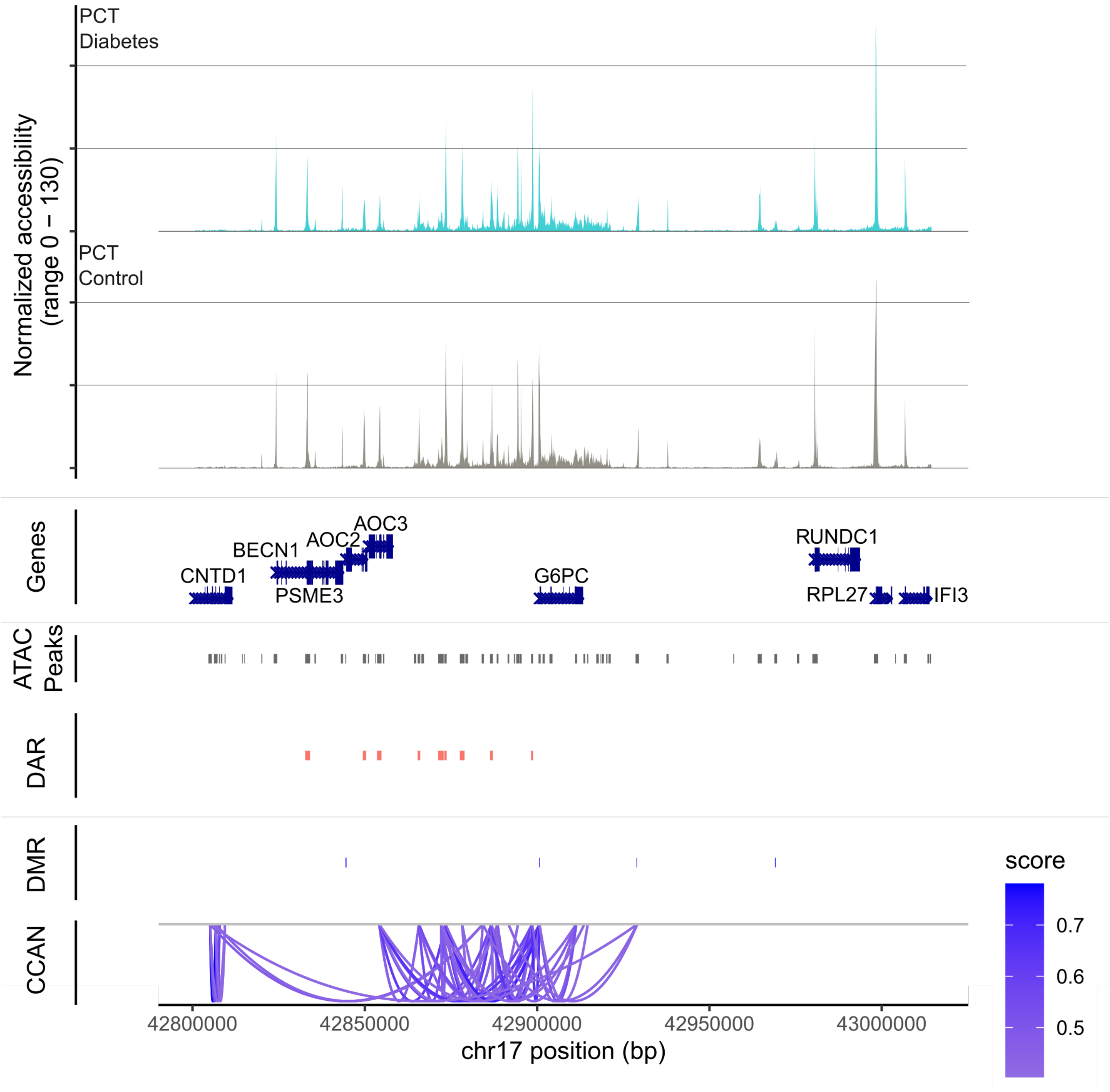

Supplemental Figure 11: Proximal tubule-specific DAR and ATAC peaks in *G6PC*. snATAC-seq coverage plots for DKD and control PCT are displayed in relation to the *G6PC* gene body. (ATAC Peaks) – accessible ATAC peaks in PCT. (DAR) – cell-specific DAR in diabetic PCT (Supplemental Data 3). (DMR) – differentially methylated regions (Supplemental Data 19) (CCAN) - Blue arcs depict the nodes of a cis-coaccessibility network (CCAN).

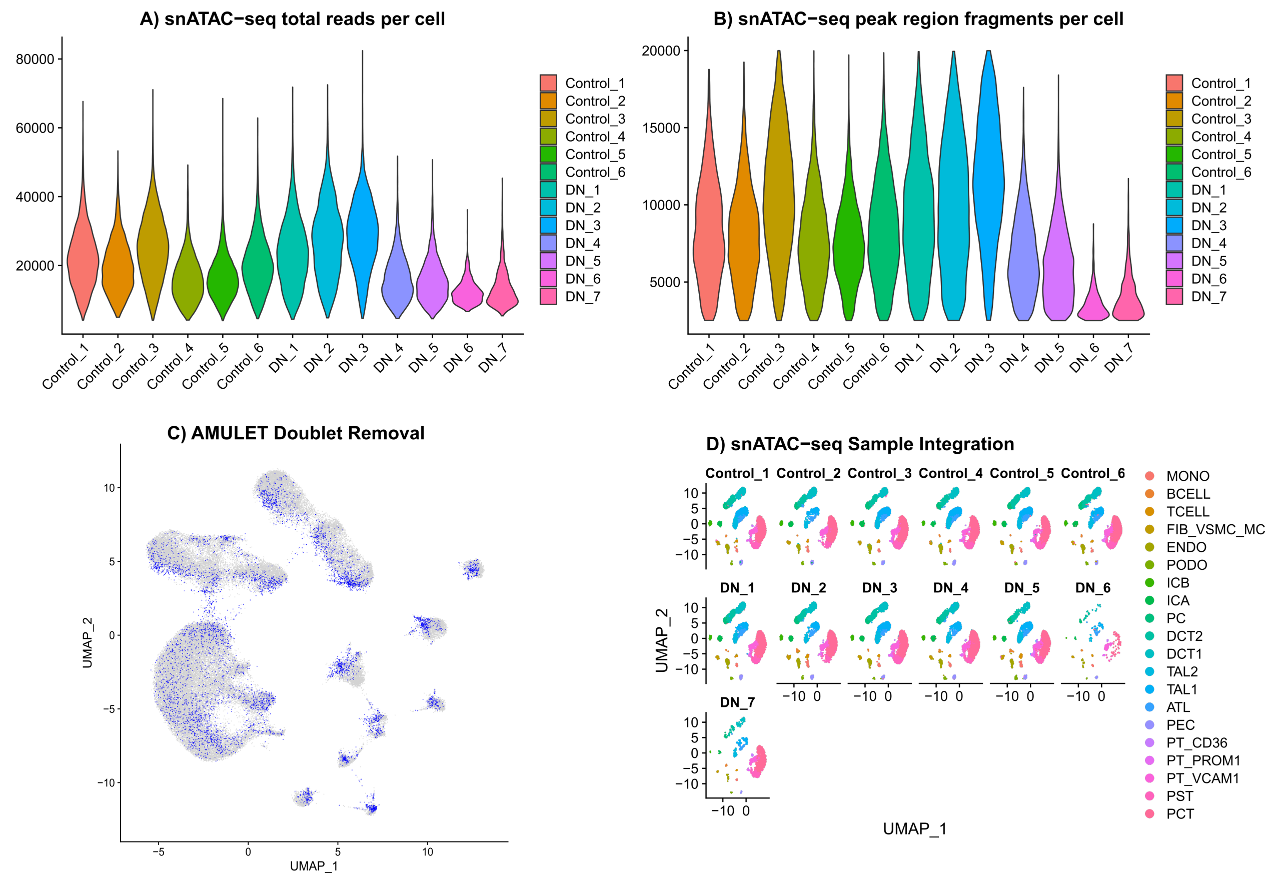

Supplemental Figure 12. snATAC-seq quality control metrics, doublet detection, and sample integration. (A) – The distribution of total reads per cell is shown for each library after quality control was completed (B) – The distribution of peak region fragments per cell is shown for each library after quality control was completed (C) – AMULET was used to detect doublets in each library and the designated doublet barcodes were visualized in the aggregated object prior to removal (D) – The distribution of cells for each library is visualized following batch effect correction with Harmony.

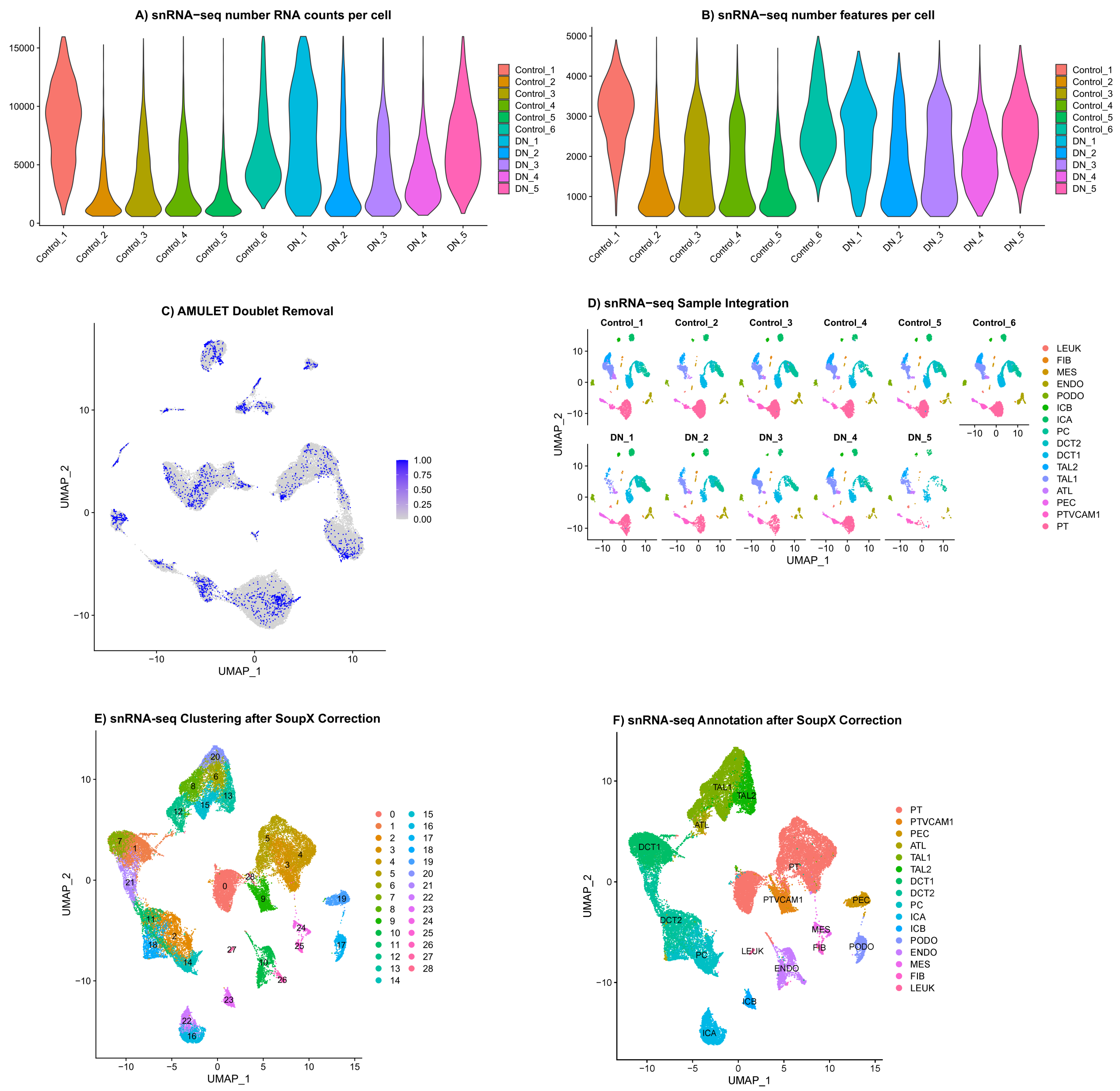

Supplemental Figure 13. snRNA-seq quality control metrics, doublet detection, sample integration, and ambient RNA correction. (A) – The distribution of total reads per cell is shown for each library after quality control was completed (B) – The distribution of features per cell is shown for each library after quality control was completed (C) – DoubletFinder was used to detect doublets in each library and the designated doublet barcodes were visualized in the aggregated object prior to removal (D) – The distribution of cells for each library is visualized following batch effect correction with Harmony. (E) – SoupX was used to correct for ambient RNA and is shown with original clustering (F) – SoupX was used to correct for ambient RNA and is shown with annotated cell types.
