# Supplementary material for: Multimodal single cell sequencing implicates chromatin accessibility and genetic background in diabetic kidney disease progression
Source: Nat Commun. 2022 Sep 6;13:5253. doi: 10.1038/s41467-022-32972-z (PMC9448792; doi:10.1038/s41467-022-32972-z)
Supplement: Supplementary file 3 — Description of Additional Supplementary Files [file 41467_2022_32972_MOESM3_ESM.docx]

**Description of Supplemental Data**

**Supplemental Data 1**

**Filename:** SD1_study_metadata.xlsx

1. metadata – Demographic, medication, laboratory, and pathology data for donor samples
2. atac_cell_distribution – Number of cells by donor and cell type in the snATAC-seq dataset
3. rna_cell_distribution – Number of cells by donor and cell type in the snRNA-seq dataset
4. snrna_qc – General quality control metrics for the snRNA-seq dataset and the total number of SNV identified by SALSA that passed filters
5. snatac_qc - General quality control metrics for the snATAC-seq dataset and the total number of SNV identified by SALSA that passed filters
6. joint_variants – Total number of variants that passed filters, were in the phased reference, and were heterozygous SNV in the combined snRNA-seq and snATAC-seq call set.

**Supplemental** **Data 2**

**Filename:** SD2_dar.macs2.celltype.markers.xlsx

**Script:** Wilson_Muto_NComm_2022/analysis/find_dar.R

Cell-specific differentially accessible regions (DAR) were identified for each cell type in the aggregated snATAC-seq dataset. Each sheet corresponds to a cell type annotation and includes the standard output from the Seurat FindMarkers function, including the ATAC peak in hg38 coordinates, the unadjusted p value (p_val), log2-fold-change for the cell type vs. other cell types (avg_log2FC), the proportion of analyzed cells containing a peak in the region (pct.1), the proportion of control cells containing a peak in the region (pct.2), the Bonferroni-adjusted Wilcoxon Rank Sum p value, the nearest protein-coding gene obtained from the Signac ClosestFeature function (gene), and the distance to this gene (distance) in bp.

**Supplemental Data 3**

**Filename:** SD3_dar.macs2.celltype.diab_vs_ctrl.xlsx

**Script:** Wilson_Muto_NComm_2022/analysis/find_dar.R

Cell-specific differentially accessible regions (DAR) were identified for each cell type in the aggregated snATAC-seq dataset by comparing DKD to control cells. Each sheet corresponds to a cell type annotation and includes the standard output from the Seurat FindMarkers function, including the ATAC peak in hg38 coordinates, the unadjusted p value (p_val), log2-fold-change for the DKD cells vs. control cells (avg_log2FC), the proportion of DKD cells containing a peak in the region (pct.1), the proportion of control cells containing a peak in the region (pct.2), the Bonferroni-adjusted Wilcoxon Rank Sum p value, the nearest protein-coding gene obtained from the Signac ClosestFeature function (gene), and the distance to this gene (distance) in bp.

**Supplemental Data 4**

**Filename:** SD4_dar.macs2.PCT_vs_PTVCAM1.markers.xlsx

**Script:** Wilson_Muto_NComm_2022/analysis/find_dar.R

Cell-specific differentially accessible regions (DAR) were identified by comparing PT_VCAM1 to PCT cells. This workbook includes the standard output from the Seurat FindMarkers function, including the ATAC peak in hg38 coordinates, the unadjusted p value (p_val), log2-fold-change for the PT_VCAM1 cells vs. PCT cells (avg_log2FC), the proportion of PT_VCAM1 cells containing a peak in the region (pct.1), the proportion of PCT cells containing a peak in the region (pct.2), the Bonferroni-adjusted Wilcoxon Rank Sum p value, the nearest protein-coding gene obtained from the Signac ClosestFeature function (gene), and the distance to this gene (distance) in bp.

**Supplemental Data 5**

**Filename:** SD5_deg.celltype.markers.xlsx

**Script:** Wilson_Muto_NComm_2022/analysis/find_deg.R

Cell-specific differentially expressed genes (DEG) were identified for each cell type in the aggregated snRNA-seq dataset. Each sheet corresponds to a cell type annotation and includes the standard output from the Seurat FindMarkers function, including the gene name, the unadjusted p value (p_val), log2-fold-change for the cell type vs. other cell types (avg_log2FC), the proportion of analyzed cells containing counts in the gene (pct.1), the proportion of control cells containing counts in the gene (pct.2), and the Bonferroni-adjusted Wilcoxon Rank Sum p value.

**Supplemental Data 6**

**Filename:** SD6_deg.celltype.diab_vs_ctrl.xlsx

**Script:** Wilson_Muto_NComm_2022/analysis/find_deg.R

Cell-specific differentially expressed genes (DEG) were identified for each cell type in the aggregated snRNA-seq dataset by comparing DKD to control cells. Each sheet corresponds to a cell type annotation and includes the standard output from the Seurat FindMarkers function, including gene name, the unadjusted p value (p_val), log2-fold-change for the DKD cells vs. control cells (avg_log2FC), the proportion of DKD cells containing counts in the gene (pct.1), the proportion of control cells containing counts in the gene (pct.2), and the Bonferroni-adjusted Wilcoxon Rank Sum p value.

**Supplemental Data 7**

**Filename:** SD7_deg.PT_vs_PTVCAM1.xlsx

**Script:** Wilson_Muto_NComm_2022/analysis/find_deg.R

Differentially expressed genes (DEG) were identified by comparing PT_VCAM1 to PCT cells. This workbook includes the standard output from the Seurat FindMarkers function, including the gene name, the unadjusted p value (p_val), log2-fold-change for the PT_VCAM1 cells vs. PCT cells (avg_log2FC), the proportion of PT_VCAM1 cells containing counts in the gene (pct.1), the proportion of PCT cells containing counts in the gene (pct.2), and the Bonferroni-adjusted Wilcoxon Rank Sum p value.

**Supplemental Data 8**

**Filename:** SD8_tcre.pool.CRE.annot.xlsx

**Script:** Wilson_Muto_NComm_2022/scafe/step2_scafe_pool.sh

Transcribed cis-regulatory elements were obtained by analyzing 2 control and 2 DKD samples sequenced with 5-prime snRNA-seq using SCAFE and pooled with scafe.workflow.sc.pool. The workbook is a pooled and annotated capped TSS file (*.ctss.bed) where coordinates are in hg38 and the transcript ID is in the 4^th^ column.

**Supplemental Table 9**

**Filename:** SD9_tcre4.celltype.diab_vs_ctrl.xlsx

**Script:** Wilson_Muto_NComm_2022/scafe/step3_scafe_prep4.R

Cell-specific differentially transcribed cis-regulatory elements (tCRE) were identified for each cell type in the aggregated SCAFE dataset by comparing DKD to control cells. Each sheet corresponds to a cell type annotation and includes the standard output from the Seurat FindMarkers function, including tCRE hg38 coordinates, the unadjusted p value (p_val), log2-fold-change for the DKD cells vs. control cells (avg_log2FC), the proportion of DKD cells containing counts in the tCRE (pct.1), the proportion of control cells containing counts in the tCRE (pct.2), and the Bonferroni-adjusted Wilcoxon Rank Sum p value.

**Supplemental Data 10**

**Filename:** SD10_tcre.PT_vs_PTVCAM1.markers.xlsx

**Script:** Wilson_Muto_NComm_2022/scafe/step3_scafe_prep4.R

Cell-specific differentially transcribed cis-regulatory elements (tCRE) were identified by comparing PT_VCAM1 to PT cells. The workbook includes the standard output from the Seurat FindMarkers function, including tCRE hg38 coordinates, the unadjusted p value (p_val), log2-fold-change for the PT_VCAM1 cells vs. PCT cells (avg_log2FC), the proportion of PT_VCAM1 cells containing counts in the tCRE (pct.1), the proportion of PCT cells containing counts in the tCRE (pct.2), and the Bonferroni-adjusted Wilcoxon Rank Sum p value.

**Supplemental Data 11**

**Filename:** SD11_kidney_GR_peaks.xlsx

**Script:** Wilson_Muto_NComm_2022/macs2/macs2.sh

Consensus control bulk kidney GR CUT&RUN peaks called with macs2 in hg38 coordinates and output in narrowPeak format. Note that consensus peaks are generated in the same manner as bulk ATAC peaks seen here: Wilson_Muto_NComm_2022/bulk_atac/step4_peakcall.sh

**Supplemental Data 12**

**Filename:** SD12_motifs_in_dar.macs2.celltype.xlsx

**Script:** Wilson_Muto_NComm_2022/analysis/find_motifs.R

Cell-specific enriched transcription factor motifs were identified for each cell type in the aggregated snATAC-seq dataset. The output was obtained using the Signac FindMotifs function and includes the motif (JASPAR2020), the number of times the motif was observed in the analyzed cell type, the number of times the motif was observed in the background cell types, the percent of observations in the analyzed cell type, the percent of observations in the background cell types, the fold enrichment in the analyzed cell type, the unadjusted p value, and the motif name. Statistical significance was determined using a hypergeometric test.

**Supplemental Data 13**

**Filename:** SD13_motifs_in_dar.macs2.celltype.diab_vs_ctrl.xlsx

**Script:** Wilson_Muto_NComm_2022/analysis/find_motifs.R

Cell-specific enriched transcription factor motifs were identified for each cell type in the aggregated snATAC-seq dataset by comparing DKD to control cells. The output was obtained using the Signac FindMotifs function and includes the motif (JASPAR2020), the number of times the motif was observed in the DKD cell type, the number of times the motif was observed in the control cell type, the percent of observations in the DKD cell type, the percent of observations in the control cell type, the fold enrichment in the DKD cell type, the unadjusted p value, and the motif name. Statistical significance was determined using a hypergeometric test.

**Supplemental Data 14**

**Filename:** SD14_chromvar.macs2.celltype.diab_vs_ctrl.xlsx

**Script:** Wilson_Muto_NComm_2022/analysis/find_motifs.R

Cell-specific enriched transcription factor activities were identified for each cell type in the aggregated snATAC-seq dataset by comparing DKD to control cells with chromVAR in Signac. The output includes the motif (JASPAR2020), the average log2 fold-change for DKD vs. control cells, the proportion of control cells with the motif, the proportion of DKD cells with the motif, the hypergeometric p value, and the gene name. Statistical significance was determined using a hypergeometric test.

**Supplemental Data 15**

**Filename:** SD15_hTERT_GR_consensus.xlsx

**Script:** Wilson_Muto_NComm_2022/macs2/macs2.sh

Consensus hTERT-RPTEC GR CUT&RUN peaks called with macs2 in hg38 coordinates and output in bed format. Note that consensus peaks are generated in the same manner as bulk ATAC peaks seen here: Wilson_Muto_NComm_2022/bulk_atac/step4_peakcall.sh

**Supplemental Data 16**

**Filename:** SD16_hTERT_ATAC_consensus.xlsx

**Script:** Wilson_Muto_NComm_2022/bulk_atac/step4_peakcall.sh

Consensus hTERT-RPTEC bulk ATAC peaks called with macs2 in hg38 coordinates and output in bed format.

**Supplemental Data 17**

**Filename:** SD17_Prim_ATAC_consensus.xlsx

**Script:** Wilson_Muto_NComm_2022/bulk_atac/step4_peakcall.sh

Consensus primary RPTEC bulk ATAC peaks called with macs2 in hg38 coordinates and output in bed format.

**Supplemental Data 18**

**Filename:** SD18_Fan_deg_bulk.xlsx

**Script:** Wilson_Muto_NComm_2022/bulk_rnaseq/find_bulk_degs.R

Raw bulk RNA-seq data was downloaded from Fan et al. PMID:31578193, counted with Salmon, and analyzed with DESeq2. Output includes the gene name, mean baseline expression, log2 fold-change for DKD vs. control, log-fold-change standard error, two-tailed Wald test statistic, unadjusted p value, and Benjamini-Hochberg adjusted p value.

Sheet: A_deg_adv_bulk – Differentially expressed genes obtained by comparing bulk RNA-seq control samples to advanced diabetic kidney disease samples.

Sheet: B_deg_early_bulk – Differentially expressed genes obtained by comparing bulk RNA-seq control samples to early diabetic kidney disease samples.

**Supplemental Data 19**

**Filename:** SD19_intersection_DMR_with_DAR_and_GR_cut_and_run.xlsx

**Script:** Wilson_Muto_NComm_2022/methylation/methylation_comparison.R

Differentially methylated regions were extracted from publicly-available databases (pmid33933144, pmid33144501, pmid31165727, pmid24098934, pmid24253112), lifted over to hg38 with the UCSC utility, flanked with a 1kb window, and intersected with regions of interest.

Sheet: ALL_DMR – A compiled list of differentially methylated regions obtained from the aforementioned studies. Seqnames, start, and end columns correspond to hg38 liftover coordinates and the dmr column corresponds to original coordinates in either hg19 or hg18 depending on the study. Note that pmid:24098934 is the only study with DMR in hg18 and all the other studies had original coordinates in hg19. The study_id column represents the PMID that the DMR was obtained from and the phenotype column describes the association between the DMR and a kidney disease phenotype.

Sheet: DAR_DKD_VS_CONTROL – The list of DMR from the ALL_DMR sheet was intersected with cell-specific DAR for DKD vs. control cells (Supplemental Table 3). The seqnames, start, and end columns correspond to the intersection between a 1kb flanked DMR and cell-specific DAR, including its width, and strand designation. (p_val) - unadjusted p-value for overlapping cell-specific DAR, (avg_log2FC) - log2-fold change for DKD vs control cells, (pct.1) - percent of control cells containing ATAC peak, (pct.2) percent of DKD cells containing ATAC peak, (p_val_adj) - Bonferroni adjusted p-value for overlapping cell-specific DAR, (gene) - nearest protein-coding gene for cell-specific DAR, (dist) - distance to the nearest gene, (celltype) - cell type corresponding to the DAR, (overlap_atac_peak) - the coordinates of the cell-specific DAR in hg38, (dmr) – original coordinates of DMR in hg18 or hg19 depending on the study, (study_id) – PMID of corresponding study DMR was obtained from, (phenotype) – DMR-associated phenotype

Sheet: Bulk_GR_CUT_AND_RUN – The list of DMR from the ALL_DMR sheet was intersected with bulk kidney GR CUT&RUN sites (Supplemental Table 11). Seqnames, start, and end columns correspond to the intersection between a 1kb flanked DMR and bulk kidney GR CUT&RUN peaks in hg38, including the width and strandedness of the intersection. (gr_peak) – hg38 coordinates of overlapping GR peak, (dmr) - original coordinates of DMR in either hg19 or hg18 depending on the study. (study_id) - PMID that the DMR was obtained from, (phenotype) - association between the DMR and a kidney disease phenotype.

Sheet: hTERT_RPTEC_GR_CUT_AND_RUN – The list of DMR from the ALL_DMR sheet was flanked with a 1kb window and intersected with hTERT RPTEC GR CUT&RUN sites (Supplemental Table 15). Seqnames, start, and end columns correspond to the intersection between a 1kb flanked DMR and hTERT-RPTEC GR CUT&RUN peaks in hg38, including the width and strandedness of the intersection. (gr_peak) – hg38 coordinates of overlapping GR peak, (dmr) - original coordinates of DMR in either hg19 or hg18 depending on the study, (study_id) - PMID that the DMR was obtained from, (phenotype) - association between the DMR and a kidney disease phenotype.

**Supplemental Data 20**

**Filename: SD20_salsa_glmer_models.xlsx**

**Script:** Wilson_Muto_NComm_2022/allele_specific/model_allele_counts.R

Modeling allele-specific chromatin accessibility with SALSA.

Sheet: fig7c_ASCA – The ratio of reference to alternate alleles in an ATAC peak was evaluated with an exact binomial test and adjusted with Benjamini Hochberg for N=11 biologically independent samples. Significance was evaluated at an adjusted p-value < 0.05.

Sheet: A_base_model - Allele-specific chromatin accessibility in an ATAC peak (binary dependent variable) was modeled as a function of imputed RNA expression (continuous predictor variable) with a mixed effect by donor using the glmer package in R. (peak) – hg38 coordinates of ATAC peak, (gene) – target gene name obtained from Signac LinkPeaks function, (estimate_exp) – log-odds estimate of expression effect size, (std.error_exp) – standard error of expression effect in log-odds, (p.value_exp) – unadjusted Wald test p value for expression effect, (conf.low_exp) – lower limit of 95% CI for expression effect, (conf.high_exp) – upper limit of 95% CI for expression effect, (estimate_sd__(Intercept)) – standard deviation of random effect intercept

Sheet: B_model2 - Allele-specific chromatin accessibility in an ATAC peak (binary dependent variable) was modeled as a function of imputed RNA expression (continuous predictor variable) and diabetes (categorical predictor variable) with a mixed effect by donor using the glmer package in R. (peak) – hg38 coordinates of ATAC peak, (gene) – target gene name obtained from Signac LinkPeaks function, (estimate_exp) – log-odds estimate of expression effect size, (std.error_exp) – standard error of expression effect in log-odds, (p.value_exp) – unadjusted Wald test p value for expression effect, (conf.low_exp) – lower limit of 95% CI for expression effect, (conf.high_exp) – upper limit of 95% CI for expression effect, (estimate_sd__(Intercept)) – standard deviation of random effect intercept, (estimate_diabetes) – log-odds estimate of diabetes effect size, (std.error_diabetes) – standard error of diabetes effect in log-odds, (p.value_ diabetes) – unadjusted Wald test p value for diabetes effect, (conf.low_diabetes) – lower limit of 95% CI for diabetes effect, (conf.high_diabetes) – upper limit of 95% CI for diabetes effect, (estimate_sd__(Intercept)) – standard deviation of random effect intercept

Sheet: C_model3 - Allele-specific chromatin accessibility in an ATAC peak (binary dependent variable) was modeled as a function of imputed RNA expression (continuous predictor variable), diabetes (categorical predictor variable), and an interaction between diabetes and expression with a mixed effect by donor using the glmer package in R. (peak) – hg38 coordinates of ATAC peak, (gene) – target gene name obtained from Signac LinkPeaks function, (estimate_exp) – log-odds estimate of expression effect size, (std.error_exp) – standard error of expression effect in log-odds, (p.value_exp) – unadjusted Wald test p value for expression effect, (conf.low_exp) – lower limit of 95% CI for expression effect, (conf.high_exp) – upper limit of 95% CI for expression effect, (estimate_sd__(Intercept)) – standard deviation of random effect intercept, (estimate_diabetes) – log-odds estimate of diabetes effect size, (std.error_diabetes) – standard error of diabetes effect in log-odds, (p.value_ diabetes) – unadjusted Wald test p value for diabetes effect, (conf.low_diabetes) – lower limit of 95% CI for diabetes effect, (conf.high_diabetes) – upper limit of 95% CI for diabetes effect, (estimate_sd__(Intercept)) – standard deviation of random effect intercept, (estimate_exp:diabetes) – log-odds estimate of interaction effect size, (std.error__exp:diabetes) – standard error of interaction effect in log-odds, (p.value_exp:diabetes) – unadjusted Wald test p value for interaction effect, (conf.low_exp:diabetes) – lower limit of 95% CI for interaction effect, (conf.high_exp:diabetes) – upper limit of 95% CI for interaction effect, (estimate_sd__(Intercept)) – standard deviation of random effect intercept

**Supplemental Data 21**

**Filename:** SD21_primers.xlsx

Sheet: qPCR_primers – Forward and reverse primer sequences for qPCR target genes for hTERT-RPTEC CRISPRi experiment validation

Sheet: sgRNA – sgRNA sequence for CRISPRi FKBP5 target regions and control non-targeting sgRNA.
