# Supplementary material for: Multimodal single cell sequencing implicates chromatin accessibility and genetic background in diabetic kidney disease progression
Source: Nat Commun. 2022 Sep 6;13:5253. doi: 10.1038/s41467-022-32972-z (PMC9448792; doi:10.1038/s41467-022-32972-z)
Supplement: Supplementary file 25 — Reporting Summary [file 41467_2022_32972_MOESM25_ESM.pdf]

nature portfolio

Corresponding author(s): Humphreys, Benjamin

Last updated by author(s): Aug 23, 2022

Reporting Summary

Nature Portfolio wishes to improve the reproducibility of the work that we publish. This form provides structure for consistency and transparency in reporting. For further information on Nature Portfolio policies, see our Editorial Policies and the Editorial Policy Checklist.

Statistics

For all statistical analyses, confirm that the following items are present in the figure legend, table legend, main text, or Methods section.

n/a	Confirmed	
☐	☒	The exact sample size (n) for each experimental group/condition, given as a discrete number and unit of measurement
☐	☒	A statement on whether measurements were taken from distinct samples or whether the same sample was measured repeatedly
☐	☒	The statistical test(s) used AND whether they are one- or two-sided<br>*Only common tests should be described solely by name; describe more complex techniques in the Methods section.*
☐	☒	A description of all covariates tested
☐	☒	A description of any assumptions or corrections, such as tests of normality and adjustment for multiple comparisons
☐	☒	A full description of the statistical parameters including central tendency (e.g. means) or other basic estimates (e.g. regression coefficient) AND variation (e.g. standard deviation) or associated estimates of uncertainty (e.g. confidence intervals)
☐	☒	For null hypothesis testing, the test statistic (e.g. F, t, r) with confidence intervals, effect sizes, degrees of freedom and P value noted<br>*Give P values as exact values whenever suitable.*
☐	☒	For Bayesian analysis, information on the choice of priors and Markov chain Monte Carlo settings
☐	☒	For hierarchical and complex designs, identification of the appropriate level for tests and full reporting of outcomes
☒	☐	Estimates of effect sizes (e.g. Cohen's d, Pearson's r), indicating how they were calculated

Our web collection on statistics for biologists contains articles on many of the points above.

Software and code

Policy information about availability of computer code

Data collection	Single nucleus RNA and ATAC datasets were aligned and counted with cellranger v4.0 and cellranger-atac v2.0.
Data analysis	Single cell datasets were analyzed in R4.1.0 using Seurat 4.0.3, harmony 0.1.0, DoubletFinder 2.0.3, AMULET 1.1.0, Signac 1.3.0, JASPAR 0.99.10, TFBSTools 1.39.0, motifmatchr 1.14.0, chromVAR 1.14.0, cicero 1.3.4.11, SCAFE 1.0, circlize (0.4.13). Heritability was partitioned with ldsc 1.0 . Single cell allele-specific counts were generated from aligned bam files using open-source code available at https://github.com/p4rkerw/SALSA . The SALSA docker container is publicly-available on Dockerhub and located at https://hub.docker.com/repository/docker/p4rkerw/salsa . The SALSA docker container and workflow relies on open-source software, including: GATK 4.2.0.0, bwa 0.7.17, STAR 2.5.1b, bcftools 1.9 , pysam 0.15.3, shapeit 4.2 and WASP 0.3.4. Single cell allele-specific counts were modeled with lme4 (1.1-27.1). CUT&RUN datasets were analyzed with trim galore (2.8), bowtie (2.3.5.1), samtools 1.9, DeepTools 3.5.0, and macs2 (2.2.7.1). Bulk RNA-seq transcript abundance was quantified with salmon (1.8.0) and analyzed with DESeq2 (1.32.0). All code for this manuscript is available at https://github.com/p4rkerw/Wilson_Muto_NComm_2022.

For manuscripts utilizing custom algorithms or software that are central to the research but not yet described in published literature, software must be made available to editors and reviewers. We strongly encourage code deposition in a community repository (e.g. GitHub). See the Nature Portfolio guidelines for submitting code & software for further information.

Data

Policy information about availability of data

All manuscripts must include a data availability statement. This statement should provide the following information, where applicable:

- Accession codes, unique identifiers, or web links for publicly available datasets
- A description of any restrictions on data availability
- For clinical datasets or third party data, please ensure that the statement adheres to our policy

All of the data for this manuscript have been made publicly-available. Raw sequencing data for snATAC-seq (N=1 control, N=7 DKD) and snRNA-seq (N=1 control, N=2 DKD) is deposited in GEO under accession number GSE195460. Previously published raw sequencing data for snRNA-seq (N=5 control, N=3 DKD) and snATAC-seq (N=5 control) are available in GEO (GSE151302, GSE131882). Processed count matrices for all snRNA-seq (N=11) and snATAC-seq (N=13) libraries used in this study are provided in GSE195460. Sequencing data for CUT&RUN from bulk kidney cortex and primary RPTEC are deposited under accession number GSE195443. Sequencing data for Omni-ATAC from hTERT-RPTEC and primary RPTEC are also deposited under accession number GSE195443. Gene expression and chromatin accessibility for each cell type can be viewed on our interactive website; Kidney Interactive Transcriptomics (http://humphreyslab.com/SingleCell) (dataset: Wilson and Muto et al.)

Field-specific reporting

Please select the one below that is the best fit for your research. If you are not sure, read the appropriate sections before making your selection.

- [x] Life sciences
- [ ] Behavioural & social sciences
- [ ] Ecological, evolutionary & environmental sciences

For a reference copy of the document with all sections, see nature.com/documents/nr-reporting-summary-flat.pdf

Life sciences study design

All studies must disclose on these points even when the disclosure is negative.

Sample size	No statistical method was used to predetermine sample size
Data exclusions	No data were excluded from the analyses
Replication	When possible, all experimental results were validated with technical replicates.
Randomization	The experiments were not randomized
Blinding	The Investigators were not blinded to allocation during experiments and outcome assessment

Reporting for specific materials, systems and methods

We require information from authors about some types of materials, experimental systems and methods used in many studies. Here, indicate whether each material, system or method listed is relevant to your study. If you are not sure if a list item applies to your research, read the appropriate section before selecting a response.

Materials & experimental systems

n/a	Involved in the study	
[ ]	[x]	Antibodies
[ ]	[x]	Eukaryotic cell lines
[x]	[ ]	Palaeontology and archaeology
[x]	[ ]	Animals and other organisms
[ ]	[x]	Human research participants
[x]	[ ]	Clinical data
[ ]	[x]	Dual use research of concern

Methods

n/a	Involved in the study	
[x]	[ ]	ChIP-seq
[x]	[ ]	Flow cytometry
[x]	[ ]	MRI-based neuroimaging

Antibodies

Antibodies used	Rabbit glucocorticoid receptor antibody [abcam, ab225886 ,1:20] and rabbit IgG negative control antibody [Epicypher, 13-0041k, 1:50])
Validation	The glucocorticoid receptor antibodies were validated in renal proximal tubule epithelial cells (RPTEC) and human bulk kidney cortex by comparing the CUT&RUN binding profiles to the accompanying IgG negative control. CUT&RUN binding sites coincided with known GR binding motifs. The concentration and duration of antibody were determined empirically to maximize assay sensitivity. ab225886 has been validated by abcam for the following applications. The application notes include recommended starting dilutions;

optimal dilutions/concentrations should be determined by the end user. Application Abreviews Notes: WB: 1/2000 - 1/10000. Predicted molecular weight: 86 kDa. IP: Use at 2-10 µg/mg of lysate. IHC-P: 1/500 - 1/2000. Perform heat mediated antigen retrieval with citrate buffer pH 6 before commencing with IHC staining protocol. ChIP-sequencing Use 4 µg for 30 µg of chromatin.

Eukaryotic cell lines

Policy information about cell lines

Cell line source(s)	RPTEC - Lonza - Catalog #: CC-2553; hTERT-RPTEC - ATCC - Catalog #: CRL-4031; HEK293 - ATCC - Catalog #: CRL-3216
Authentication	Proximal tubule cell lines were authenticated by expression of expected proximal tubule markers
Mycoplasma contamination	All cell lines were negative for mycoplasma contamination
Commonly misidentified lines (See ICLAC register)	*Name any commonly misidentified cell lines used in the study and provide a rationale for their use.*

Human research participants

Policy information about studies involving human research participants

Population characteristics	For snRNA-seq and snATAC-seq, a total of thirteen kidney cortex samples were obtained from control patients (n=6), and patients with diabetic kidney disease (DKD, n=7). Tissue samples were collected following nephrectomy for renal mass (n=10) or from deceased organ donors (n=3). Patients ranged in age from 50 to 78 years (median=57y) and included seven men and six women (Supplemental Table 1). Patients with type 2 diabetes had elevated hemoglobin A1c (mean=8.2 +/- 1.5%). The majority of patients with DKD were on antihypertensive or ACE inhibitor therapy and two patients were on insulin. Two patients with DKD had mild to moderate proteinuria as measured by urine dipstick. Tissue sections were stained with H&E and examined by a renal pathologist (P.W.) to evaluate histological features of DKD. Control samples did not have significant global glomerulosclerosis (<10%) or interstitial fibrosis and tubular atrophy (<10%). Patients with DKD had predominantly mild (N=3, < 25%) or moderate (N=3, 26-50%) global glomerulosclerosis with a corresponding increase in interstitial fibrosis and tubular atrophy (Supplemental Figure 1). Mean eGFR of DKD samples (66 +/- 25 ml/min/1.73m^2) and control samples (74 +/- 15ml/min/1.73m^2) was not statistically different (Students t-test, p=0.49). DKD samples showed nodular mesangial expansion, thickened glomerular basement membranes and afferent arteriolar hyalinosis.
Recruitment	For snRNA-seq and snATAC-seq, non-tumor kidney cortex samples (n=10) were obtained from patients undergoing partial or radical nephrectomy for renal mass at Brigham and Women's Hospital (Boston, MA) under an established Institutional Review Board protocol approved by the Mass General Brigham Human Research Committee. An additional three kidney cortex samples (1 control and 2 DKD) were obtained from deceased organ donors in the Novo Nordisk biorepository. For bulk kidney GR CUT&RUN and Omni-ATAC, kidney cortex samples were obtained from deceased organ donors (N=3).
Ethics oversight	Mass General Brigham Human Research Committee, Washington University Institutional Review Board

Note that full information on the approval of the study protocol must also be provided in the manuscript.

Dual use research of concern

Policy information about dual use research of concern

Hazards

Could the accidental, deliberate or reckless misuse of agents or technologies generated in the work, or the application of information presented in the manuscript, pose a threat to:

No	Yes	
☒	☐	Public health
☒	☐	National security
☒	☐	Crops and/or livestock
☒	☐	Ecosystems
☒	☐	Any other significant area

Experiments of concern

Does the work involve any of these experiments of concern:

No	Yes	
☒	☐	Demonstrate how to render a vaccine ineffective
☒	☐	Confer resistance to therapeutically useful antibiotics or antiviral agents
☒	☐	Enhance the virulence of a pathogen or render a nonpathogen virulent
☒	☐	Increase transmissibility of a pathogen
☒	☐	Alter the host range of a pathogen
☒	☐	Enable evasion of diagnostic/detection modalities
☒	☐	Enable the weaponization of a biological agent or toxin
☒	☐	Any other potentially harmful combination of experiments and agents
